# Adiponectin Gene Polymorphism rs2241766 T/G Is Associated with Response to Pioglitazone Treatment in Type 2 Diabetic Patients from Southern China

**DOI:** 10.1371/journal.pone.0112480

**Published:** 2014-11-18

**Authors:** Hong Yang, Enling Ye, Guangxin Si, Liangmiao Chen, Lingqiao Cai, Chengfu Ye, Chi Zhang, Xuemian Lu

**Affiliations:** 1 Department of Endocrinology, the Third Hospital Affiliate to Wenzhou Medical University, Ruian, Zhejiang, China; 2 Ruian Center of the Chinese-American Research Institute for Diabetic Complications, the Third Affiliated Hospital of the Wenzhou Medical University, Wenzhou, China; 3 Department of Endocrinology, Shangdong Jining No. 1 People's Hospital, Jining, Shangdong, China; University of Warwick – Medical School, United Kingdom

## Abstract

**Introduction:**

Insulin sensitizing drugs such as pioglitazone are not uniformly treatment effective among individual type 2 diabetic patients. Here, the relationship of pioglitazone efficacy to single nucleotide polymorphisms (SNP) of the adiponectin gene, a critical gene directly regulated by the drug, was examined in a cohort of Chinese Han type 2 diabetic patients.

**Methods:**

Eighty type 2 diabetic patients were treated with pioglitazone (15 mg/day) for 12 weeks without interruption of their current therapeutic regimen. Fasting plasma glucose, fasting insulin, homeostasis model assessment for insulin resistance (HOMA-IR), and glycated hemoglobin (HbA1c%) were collected both prior to and following pioglitazone treatment. Response to pioglitazone was defined as a decrease of at least 15% in HbA1c% levels. Three regions of the adiponectin gene containing SNPs (promoter, intron 2 and exon 2, and exon 3) were amplified and sequenced to determine genotype.

**Results:**

Serum adiponectin levels were significantly increased (*p*<0.001) whereas fasting plasma glucose, fasting insulin, HOMA-IR, and HbA1c% values were significantly decreased relative to baseline measurements (*p*<0.001). Response of patients with TG and TT genotypes at rs2241766 (exon2; 52.9% vs. 12.7%, respectively *p* = 0.001) was statistically significant relative to all other patients. Amongst rs2241766 TG and TT patients, the mean decrease in HbA1c% levels was greater where the genotype was TG (1.15±0.80 vs. 0.52±0.64, *p* = 0.001).

**Conclusions:**

The adiponectin gene polymorphism rs2241766 T/G is associated with pioglitazone efficacy in type 2 diabetic patients, and status of the polymorphism may be an important clinical factor to consider prior to pioglitazone treatment.

## Introduction

Type 2 diabetes mellitus (T2DM) is on the rise in current populations worldwide due to changes in weight, for example. Alternative therapeutic strategies are necessary, as patients are known to develop resistance to treatment with insulin. A breakthrough in strategies for these patients occurred when it was discovered that adipocytes secrete a protein, adiponectin (ADIPOQ), which plays a role in in the regulation of glucose and lipid metabolism [1]. ADIPOQ is encoded by one of the most abundant adipose gene transcripts [2]. Regulation of the gene is known to occur at least in part through the nuclear receptor transcription factor, peroxisome proliferator activated receptor γ (PPARγ) [3]. Pharmacological compounds, such as the thiazolidinediones (TDZ), that target PPARγ activity have thus been exploited as insulin sensitizers, and are now widely used for the treatment of insulin resistance in T2DM patients.

Pioglitazone is a TZD compound that is currently used as an insulin-sensitizing drug. However, many clinical trials have shown that the therapeutic efficacy of pioglitazone is inconsistentamong individual T2DM patients [4], [5]. One explanation is underlying genetic variationas genetic mutation has been shown to be the basis for some metabolic syndromes. A number of studies have demonstrated that subtle changes in DNA sequence, single nucleotide polymorphisms (SNP), are often associated with differences in drug efficacy and toxicity among patients [6]. In recent years, many SNPs in ADIPOQ, have been linked to body mass index (BMI), insulin sensitivity, and T2DM in some cross-sectional studies [4], [7]–[9]. However, relatively few studies have addressed whether *ADIPOQ* gene polymorphisms are associated with pioglitazone therapeutic efficacy. The results so far have not been consistent. In a study with 113 T2DM Chinese patients, the rs266729 polymorphism was associated with response to pioglitazone, whereas in an Iranian population, the rs2241766 and rs16928751 polymorphisms were not related to response [10], [11]. In the current study, the association of *ADIPOQ* gene polymorphisms with pioglitazone therapeutic efficacy was investigated in Chinese Han patients with T2DM.

## Materials and Methods

### Ethics statement

The study protocol was approved by the ethics committee of the Third Affiliated Hospital of Wenzhou Medical University. The 80 unrelated individuals were long-term residents in the Wenzhou area of China, and all patients signed the informed consent prior to the study.

### Patients

Data were collected from a total of 80 outpatients with T2DM. All patients were derived from the Chinese Han population treated at the Department of Endocrinology of the Third Affiliated Hospital of Wenzhou Medical University between the years 2010 to 2012. Patients included 32 men and 48 women whose ages ranged from 34 to 80 yr (mean 57.05±10.48 yr). T2DM was diagnosed according to the World Health Organization criteria from 1999: (1) random plasma glucose≥11.1 mmol/L, fasting plasma glucose (FPG) ≥7.0 mmol/L, or 2-hour oral glucose tolerance test result was ≥11.1 mmol/L; (2) symptomless: repeat the test a second time on a different day to confirm the diagnosis; (3) any stress (due to infection, trauma, or condition) that causes the level of blood glucose to temporarily rise was excluded. The inclusion criteria were as follows: (1) FPG between 7.0 and 14.0 mmol/L and glycated hemoglobin (HbA1c%) between 7.0 and 11.5%; (2) no change in antidiabetic agents in the last three months; (3) no TZD medications of any type in the past year; and (4) no pregnancy within the past year. Patients were excluded on the basis of the following criteria: (1) type 1 diabetes; (2) a history of diabetic ketoacidosis or acute complications of diabetes, such as hyperosmolar hyperglycemic state; (3) ischemic heart disease or heart failure class II to IV (New York Heart Association; NYHA); (4) insulin treatment; and (5) pregnancy or nursing.

### Clinical measurements

Patients took 15 mg/day pioglitazone orally for 12 weeks without interruption of their current therapeutic regimen for T2DM. Venous blood samples were collected from patients after fasting overnight. Clinical and biochemical data, including age, height, weight, waist circumference (WC), blood pressure, fasting plasma glucose (FPG), fasting insulin, HbA1c%, total cholesterol (TC), triglycerides (TG), high-density lipoprotein cholesterol (HDL-C), low-density lipoprotein cholesterol (LDL-C), and ADIPOQ were collected from all 80 patients. FPG, TC, HDL-C, LDL-C, and TG were determined with the Olympus AU 5400 Automatic Biochemical Analyzer (Olympus Corporation, Mishama, Japan). Serum insulin levels were determined with the Beckman Uncicel DxI 800 Chemiluminescence Analyzer (Beckman-Coulter, Fullerton, CA). Serum ADIPOQ levels were detected by a double antibody sandwich ELISA (Shanghai Westang Bio-tech Co., Ltd.; Shanghai, China). The insulin resistance (IR) index was assessed by homeostasis model assessment (HOMA). The following formula was used to calculate the IR index: HOMA-IR  =  (FPI×FPG)/22.5 where 22.5 is a constant.

### Efficacy evaluation

Clinical samples were analyzed, and "effectiveness" was defined as a decrease in the HbA1c% level by more than 15% relative to the baseline measurement (prior to piaglitazone treatment) in individual patients.

### Genotyping analyses

Genomic DNA was isolated from patient blood with a DNA extraction kit according to the manufacturer's protocol (Shanghai Generay Biotech Co., Ltd.; Shanghai, China). Adiponectin gene SNPs were amplified by polymerase chain reaction (PCR). Primer pairs (Shanghai Generay Biotech Co., Ltd.) were designed using Primer Premier 5 and Oligo 6.0 software to amplify three regions of the adiponectin gene containing 13 polymorphisms (rs266729, rs16861194, rs1501299, rs2241767, rs3821799, rs145335235, rs3774261, rs2241766, rs17846872, rs35469083, rs1063537, rs2082940, rs1063538): promoter, upstream of exon 2 and intron 2, and upstream of intron 3 (Table 1). The reactions were performed in a total volume of 25 µl containing 0.1-1ug DNA, 0.8 µl of each primer (10 µmol/L), and 12.5 µl 2 × PCR Master Mix (Thermo Scientific; Pittsburgh, PA, USA). The cycling parameters for PCR were the following: denaturation 94°C 4 min, followed by 30 cycles of denaturation 94°C 40 s, annealing 56°C 35 s, and extension 72°C 40 s. Specificity of PCR was confirmed by 5% polyacrylamide gel electrophoresis, and products were commercially sequenced (Shanghai Sangon Biological Engineering Technology & Services Co., Ltd.; Shanghai, China).

**Table 1:** Polymerase chain reaction primers and the amplified products for adiponectin gene polymorphisms.

Region	Primer sequence	Fragment
Promoter	F:5′-TGGTGGACTTGACTTTACTGGT-3′	563 bp
	R:5′-GCTTGTGGCCTCGAATCGTA-3′	
Intron 2 and exon 2	F:5′-TGCTGTTGCTGGGAGCTGTTCT-3′	738 bp
	R:5′-CATTCTTCATCAGGTCCACGGT-3′	
Exon 3	F:5′-TGAATCCCATATCTACCC-3′	609 bp
	R:5′-TCATGCCACTGCACTCTA-3′	

F: Forward primer; R: Reverse primer.

### Statistical analysis

Statistical analyses were performed with SPSS software Version 16.0 for Windows (SPSS Inc.; Chicago, IL, USA). Data showing a normal distribution were summarized as the mean ± standard deviation (SD), and data showing a non-normal distribution were summarized as medians (P25%–P75%). Data with a normal distribution were compared with a Student's t-test; data with non-normal distributions were compared with the Wilcoxon rank-sum test. Numeration data were presented as a frequency and compared using the chi-square test. The relationship between the therapeutic efficacy of pioglitazone and individual polymorphic variants of the adiponectin gene was analyzed using the chi-square test. Logistic regression analysis of relevant variables was performed to identify predictors for therapeutic efficacy of pioglitazone. A value of *p*<0.05 was considered to be statistically significant.

## Results

### Clinical characteristics are regulated by pioglitazone

The clinical characteristics, and anthropometric and metabolic findings in the patients prior to and following 12 weeks of pioiglitazone treatment are presented in Table 2. Based on critical clinical parameters, pioglitazone appeared to have an overall effect on this cohort of Chinese Han patients. The primary measure of pioglitazone efficacy was that the change in serum ADIPOQ concentration significantly increased from 3.42 mg/l to 5.67 mg/l (*p*<0.001). Other critical clinical correlates (FPG, fasting insulin, HbA1c% levels and HOMA-IR) of the 80 patients significantly decreased with treatment (8.73±1.55 mmol/l, 6.85±3.86 mg/ml, 7.65% and 2.29 respectively vs. 7.25±1.36 mmol/l, 5.38±2.59 mg/ml, 7.10% and 1.51 respectively; *p*<0.001). Lower levels of FPG, fasting insulin, HOMA-IR, and HbA1c%, in combination with higher serum ADIPOQ concentration indicated that pioglitazone had beneficial effects on the patients. Other clinical indicators (TC, TG, LDL-C and HDL-C) did not change significantly when examined as a group; neither did weight, BMI, WC, and systolic or diastolic blood pressures.

**Table 2:** Clinical characteristics of subjects before and after pioglitazone treatment.

	Before	After	p-value
Fasting plasma glucose (mmol/L)	8.73±1.55	7.25±1.36	<0.001*
Adiponectin (mg/L)	3.42 [2.19,7.21]	5.67 [2.83,10.14]	<0.001*
Fasting insulin(mg/ml)	6.85±3.86	5.38±2.59	<0.001*
HOMA-IR	2.29 [1.61,3.28]	1.51 [1.08,2.16]	<0.001*
HbA1c(%)	7.65 [7.30,8.40]	7.10 [6.70,7.95]	<0.001*
TG(mmol/L)	1.70 [1.20,2.37]	1.52 [1.19,2.00]	0.478
TC(mmol/L)	4.73±0.87	4.72±0.96	0.955
LDL-C(mmol/L)	2.87±0.80	2.81±0.84	0.460
HDL-C(mmol/L)	1.28±0.29	1.33±0.32	0.052
Weight (Kg)	66.13±9.88	66.43±9.81	0.184
BMI(Kg/m2)	25.49±3.05	25.61±3.09	0.163
Waist circumference(cm)	90.65±8.83	90.19±8.60	0.229
Systolic blood pressure(mmHg)	133.98±20.74	132.34±17.93	0.386
Diastolic blood pressure (mmHg)	80.00 75.25,90.00]	80.00 [70.00,89.00]	0.254

HOMA-IR, homeostasis model assessment for insulin resistance; HbA1c, glycated hemoglobin; TG, triglyceride; TC, total cholesterol; LDL-C, low-density lipoprotein cholesterol; HDL-C, high-density lipoprotein cholesterol; BMI, body mass index.

### Genotyping

Thirteen SNPs of the *ADIPOQ* gene were genotyped in this study. The distribution of all genotypes (homozygous and heterozygous polymorphic variants) and allelic frequencies are shown in Table 3. All SNPs were found to be in Hardy-Weinberg equilibrium (*p*>0.05). However, three SNPs (rs35469083, rs17846872 and rs145335235) were eliminated from further analysis due to a low frequency in the target patient population.

**Table 3:** SNP genotypes of the adiponectin gene and allele frequencies in the Chinese cohort.

db SNP number	Gene region	Alleles (1/2)	Genotype distribution		
			11	12	22
rs266729	Promoter	C/G	70%	26.25%	3.75%
rs16861194	Promoter	A/G	85%	15%	0.00%
rs1501299	Intron 2	G/T	45%	46.25%	8.75%
rs2241767	Intron 2	A/G	55%	41.25%	3.75%
rs3821799	Intron 2	T/C	38.75%	53.75%	7.50%
rs145335235	Intron 2	C/T	97.50%	2.50%	0.00%
rs3774261	Intron 2	A/G	37.50%	50%	12.50%
rs2241766	Exon 2	T/G	78.75%	21.25%	0.00%
rs17846872	Exon 3	G/T	91.25%	8.75%	0.00%
rs35469083	Exon 3	C/T	97.50%	0.00%	2.50%
rs1063537	Exon 3	C/T	57.50%	30%	12.50%
rs2082940	Exon 3	C/T	47.50%	33.75%	18.75%
rs1063538	Exon 3	T/C	30%	51.25%	18.75%

### Effectiveness of pioglitazone treatment

In general, HbA1c concentrations are used as the definitive measure for whether a drug has any health benefits for patients. Any decrease by greater than 15% is defined as a response to pioglitazone. After 12 weeks of pioglitazone treatment, 21.25% of the 80 patients achieved a decrease in HbA1c% of ≥15%.

### rs2241766 is independently associated with response to pioglitazone

In general, pioglitazone appeared to be beneficial to patients. However, the major objective was to determine whether the overall efficacy of pioglitazone could be attributed to any specific subgroups of patients. Patients were, thus, grouped according to their genotype, and the change in HbA1c% levels was determined. The patients exhibiting the greatest response to 12 weeks of treatment with pioglitazone were in the group with the rs2241766 (exon 2) TG genotype (52.9%). A second rs2241766 genotype, TT, was also associated with an increased response to pioglitazone within this timeframe (12.7%). The difference in the response of patients between rs2241766 genotype TG and TT was statistically significant (*p* = 0.001). No other associations between individual *ADIPOQ* gene polymorphisms and response to pioglitazone treatment were found (Table 4). Finally, logistic regression analysis revealed that rs2241766 was independently associated with response to pioglitazone. Patients carrying the TG genotype responded significantly better in general to pioglitazone compared to the patients carrying the TT genotype (OR  = 11.483, 95% CI  = 2.637–50.002, *p* = 0.001; Table 5).

**Table 4:** Association of adiponectin gene SNP with response to pioglitazone.

SNP	Response rate	P-value (χ^2^)	SNP	Response rate	P-value (χ^2^)
Rs1501299G/T			Rs2241767A/G		
GG	16.67%		AA	27.27%	
GT+TT	25%	0.365	AG+GG	13.89%	0.145
Rs2241766T/G			Rs2082940C/T		
TG	52.94%		CC	26.32%	
TT	12.70%	0.001*	CT+TT	16.67%	0.292
Rs266729C/G			Rs1063538T/C		
CC	21.43%		TT	20.83%	
CG+GG	20.83%	0.952	TC+CC	21.43%	0.952
Rs16861194A/G			Rs1063537C/T		
AA	20.59%		CC	26.09%	
AG	25%	0.711	CT+TT	14.71%	0.219
Rs3821799T/C			Rs3774261A/G		
TT	19.35%		AA	16.67%	
TC+CC	22.45%	0.742	AG+GG	24%	0.438

**Table 5:** Logistic regression analysis for the influence of clinical and genetic factors on the response to pioglitazone treatment.

Variable	Odds ratio	95% CI	P-value
Rs1501299 (GT+TT vs. GG)	1.718	0.408–7.230	0.461
Rs2241767 (AA vs. AG+GG)	1.201	0.236–6.120	0.826
Rs3821799 (TC+CC vs. TT)	1.131	0.139–9.204	0.908
Rs3774261 (AG+GG vs. AA)	1.209	0.156–9.352	0.856
Rs266729 (CC vs. CG+GG)	1.219	0.307–4.836	0.778
Rs16861194 (AG vs. AA)	1.274	0.231–7.019	0.781
Rs1063537 (CC vs. CT+TT)	2.972	0.492–17.947	0.235
Rs2082940 (CC vs. CT+TT)	1.303	0.308–5.515	0.719
Rs1063538 (TC+CC vs. TT)	1.255	0.283–5.562	0.765
Rs2241766 (TG vs. TT)	11.483	2.637–50.002	0.001*

### Mean changes in clinical characteristics before and after pioglitazone treatment are statistically significant between genotypes

The previous analysis was based on the number of patients, out of 80 total or within genotype subgroups, with ≥15% change in HbA1c%. In order to gain further insight into the physiological benefits of pioglitazone to patients, genotype was examined with regard to each clinical parameter in terms of mean change between the measurements made prior to and following treatment. Polymorphisms at rs2241766 exhibited statistically significant changes within clinical correlates in response to treatment that were not apparent in the previous analysis. The mean decrease in HbA1c% levels after pioglitazone treatment was greater in patients with the rs2241766 genotype TG than the patients with genotype TT (TG: 1.15±0.80% vs. TT: 0.52±0.64%, *p* = 0.001), indicating a synergy of the G allele (rs2241766) with efficacy of pioglitazone in these patients. There were no other significant differences of mean change in FPG, fasting insulin, HOMA-IR, ADIPOQ, TG, TC, LDL-C, HDL-C, BMI, and WC between rs2241766 TG and TT genotypes (Table 6).

**Table 6:** Clinical characteristics of patients with various genotypes for the rs2241766 before and after the administration of pioglitazone.

		rs2241766		P-value
		TT	TG	
FPG (mmol/L)	Before treatment	8.62±1.52	9.17±1.61	0.195
	After treatment	7.22±1.43	7.40±1.07	0.637
	Mean change	1.40±1.88	1.77±1.21	0.440
Fasting insulin (mg/ml)	Before treatment	6.87±4.14	6.81±2.67	0.959
	After treatment	5.24±2.62	5.89±2.51	0.362
	Mean change	1.14 [−0.02,2.51]	0.71 [0.07,1.35]	0.473
HbA1c (%)	Before treatment	7.60 [7.20,8.20]	7.90 [7.30,8.95]	0.256
	After treatment	7.41±0.94	7.12±1.05	0.286
	Mean change	0.52±0.64	1.15±0.80	0.001*
HOMA-IR	Before treatment	2.16 [1.46,3.26]	2.45 [1.94,3.39]	0.298
	After treatment	1.47 [1.06,2.02]	1.83 [1.22,2.39]	0.137
	Mean change	0.66 [0.11,1.23]	0.77 [0.34,1.16]	0.663
Adiponectin (mg/L)	Before treatment	4.51±3.92	6.07±3.64	0.145
	After treatment	6.63±5.07	8.48±5.03	0.185
	Mean change	−2.11±3.95	−2.41±5.19	0.800
TG (mmol/l)	Before treatment	1.84±0.95	2.14±0.90	0.249
	After treatment	1.46 [1.16,2.19]	1.62 [1.31,1.86]	0.394
	Mean change	0.01±0.78	0.28±0.106	0.245
TC (mmol/l)	Before treatment	4.65±0.85	5.02±0.91	0.120
	After treatment	4.72±1.00	4.74±0.87	0.955
	Mean change	−0.07±0.90	0.28±0.90	0.153
LDL-C (mmol/l)	Before treatment	2.84±0.83	2.99±0.69	0.479
	After treatment	2.81±0.87	2.81±0.70	0.998
	Mean change	0.03±0.75	0.18±0.70	0.447
HDL-C (mmol/l)	Before treatment	1.29±0.29	1.26±0.31	0.667
	After treatment	1.35±0.34	1.26±0.23	0.261
	Mean change	−0.06±0.22	0.00±0.23	0.297
BMI (kg/m2)	Before treatment	25.63±3.28	24.94±1.96	0.276
	After treatment	25.76±3.34	25.05±1.86	0.253
	Mean change	−0.13±0.83	−0.11±0.65	0.930
Waist circumference (cm)	Before treatment	91.41±9.43	87.82±5.43	0.049*
	After treatment	90.91±9.04	87.53±6.20	0.151
	Mean change	0.00 [−1.00,2.00]	0.00 [−2.00,1.25]	0.609

FPG, fasting plasma glucose; HOMA-IR, homeostasis model assessment for insulin resistance; HbA1c, glycated hemoglobin; TG, triglyceride; TC, total cholesterol; LDL-C, low-density lipoprotein cholesterol; HDL-C, high-density lipoprotein cholesterol; BMI, body mass index.

The second clinical correlate to emerge as a potential predictor of pioglitazone efficacy was WC. Genotypes of two SNPs within the intron 2 region of the *ADIPOQ* gene (rs3774261 and rs3821799) were associated with significant differences in WC. Patients of the rs3774261 genotypes AA and AG + GG experienced a significant mean decrease of WC (AA: 1.00 [−0.63, 3.00] vs. AG + GG; 0.00 [−1.00, 1.00]; *p* = 0.019). The mean decrease in the insulin value, however, was less for genotype AA than for genotypes AG + GG (AA: 0.45 [−0.24, 1.67]; AG + GG: 1.44 [0.33, 2.52]; *p* = 0.030). For the second intron 2 polymorphism (rs3821799), the mean decrease in WC was significantly greater in those patients with genotype TT than in those with the other genotypes (TT: 1.23±2.59 cm; TC+CC: −0.03±2.39 cm; *p* = 0.033; Table 7). No significant differences in the mean change of FPG, fasting insulin, HbA1c%, HOMA-IR, ADIPOQ, TG, TC, LDL-C, HDL-C, BMI, or WC after pioglitazone treatment were found when rs2241767 and rs1501299 genotypes within the intron 2 region of *ADIPOQ* gene were examined (Table 8).

**Table 7:** Clinical characteristics of patients with various genotypes for the rs3821799 and rs3774261 before and after the administration of pioglitazone.

		rs3821799		p-value	rs3774261		p-value
		TT	TC+CC		AA	AG+GG	
FPG (mmol/L)	Before treatment	8.63±1.48	8.81±1.60	0.622	8.57±1.49	8.83±1.59	0.469
	After treatment	7.09±1.43	7.36±1.32	0.383	6.88±1.27	7.49±1.37	0.052
	Mean change	1.54±1.88	1.44±1.69	0.810	1.70±1.78	1.35±1.75	0.396
Fasting insulin (mg/ml)	Before treatment	6.02±2.95	7.38±4.29	0.125	6.11±2.95	7.30±4.29	0.183
	After treatment	5.10±2.01	5.56±2.91	0.408	5.31±2.29	5.42±2.78	0.847
	Mean change	0.59 [−0.21,1.64]	1.25 [0.32,2.54]	0.076	0.45 [−0.24,1.67]	1.44 [0.33,2.52]	0.030*
HbA1c (%)	Before treatment	8.20±1.34	7.88±0.82	0.227	8.17±1.30	7.90±0.88	0.322
	After treatment	7.47±1.12	7.27±0.86	0.374	7.49±1.15	7.26±0.83	0.345
	Mean change	0.74±0.68	0.61±0.74	0.435	0.68±0.74	0.64±0.71	0.820
HOMA-IR	Before treatment	2.16 [1.46,3.02]	2.43 [1.71,3.43]	0.187	2.06 [1.44,3.07]	2.44[1.68,3.37]	0.229
	After treatment	1.47 [1.06,1.86]	1.69 [1.09,2.33]	0.477	1.63±0.87	1.77 ±0.87	0.491
	Mean change	0.41 [0.21,0.82]	0.80 [0.30,1.27]	0.142	0.45 [0.19,0.92]	0.79[0.29,1.24]	0.178
Adiponectin (mg/L)	Before treatment	3.15 [2.68,5.53]	4.14 [1.80,7.43]	0.726	3.06 [2.67,5.75]	4.29 [1.87,7.41]	0.773
	After treatment	6.96±4.82	7.06±5.29	0.935	7.12±5.59	6.96 ±4.81	0.897
	Mean change	−2.48±4.47	−1.99±4.07	0.615	−2.49±4.93	−1.99±3.75	0.604
TG (mmol/l)	Before treatment	1.84±0.93	1.94±0.96	0.642	1.85±0.95	1.94±0.95	0.685
	After treatment	1.46 [1.05,1.90]	1.55 [1.27,2.29]	0.186	1.61 [1.18,2.07]	1.48[1.22,2.06]	0.917
	Mean change	0.20±0.79	−0.01±0.88	0.286	0.10±0.73	0.05±0.92	0.778
TC (mmol/l)	Before treatment	4.79±0.83	4.69±0.89	0.595	4.89±0.73	4.63±0.93	0.199
	After treatment	4.60±1.01	4.80±0.93	0.365	4.66±1.01	4.76±0.95	0.649
	Mean change	0.19±0.86	−0.11±0.92	0.138	0.23±0.77	−0.13±0.96	0.084
LDL-C (mmol/l)	Before treatment	2.96±0.86	2.82±0.76	0.425	3.01±0.81	2.79±0.79	0.220
	After treatment	2.84±1.04	2.79±0.69	0.807	2.87±1.03	2.78±0.71	0.636
	Mean change	0.12±0.73	0.02±0.75	0.561	0.15±0.67	0.01±0.78	0.434
HDL-C (mmol/l)	Before treatment	1.24±0.31	1.31±0.28	0.310	1.29±0.29	1.28±0.30	0.870
	After treatment	1.30±0.30	1.35±0.33	0.460	1.32±0.31	1.34±0.33	0.830
	Mean change	−0.06±0.16	−0.04±0.25	0.749	−0.03±0.13	−0.06±0.26	0.540
BMI (kg/m^2^)	Before treatment	25.30±2.83	25.60±3.20	0.666	25.64±3.40	25.40±2.85	0.734
	After treatment	25.34±2.84	25.78±3.25	0.536	25.59±3.24	25.62±3.03	0.957
	Mean change	−0.04±0.90	−0.18±0.71	0.446	0.05±0.92	−0.23±0.69	0.126
Waist circumference(cm)	Before treatment	89.71±7.96	91.24±9.37	0.452	91.38±9.62	90.21±8.39	0.568
	After treatment	88.48±8.30	91.28±8.69	0.158	90.18±9.65	90.20±8.00	0.993
	Mean change	1.23±2.59	−0.03±2.39	0.033*	1.00 [−0.63,3.00]	0.00 [−1.00,1.00]	0.019*

FPG, fasting plasma glucose; HOMA-IR, homeostasis model assessment for insulin resistance; HbA1c, glycated hemoglobin; TG, triglyceride; TC, total cholesterol; LDL-C, low-density lipoprotein cholesterol; HDL-C, high-density lipoprotein cholesterol; BMI, body mass index.

**Table 8:** Clinical characteristics of patients with various genotypes for the rs1501299 and rs2241767 before and after the administration of pioglitazone.

		rs1501299		p-value	rs2241767		p-value
		GG	GT+TT		TT	TG	
FPG (mmol/L)	Before treatment	8.35±1.11	9.05±1.78	0.034*	8.74±1.3	8.74±1.81	0.993
	After treatment	7.10±1.29	7.39±1.42	0.342	7.30±1.37	7.21±1.36	0.779
	Mean change	1.25±1.43	1.67±1.98	0.283	1.44±1.70	1.53±1.85	0.834
Fasting insulin (mg/ml)	Before treatment	6.44±2.94	7.20±4.49	0.366	7.10±3.92	6.55±3.83	0.534
	After treatment	5.33 3.69,7.00]	4.46 [3.16,6.92]	0.498	5.65±2.57	5.05±2.63	0.302
	Mean change	0.67 [−0.03,1.89]	1.11 [0.04,2.87]	0.442	0.88 [−0.04,1.89]	1.23 [0.31,2.37]	0.505
HbA1c (%)	Before treatment	7.40 [7.10,7.80]	8.10 [7.4,8.90]	0.001*	8.19±1.12	7.77±0.94	0.073
	After treatment	7.07±0.76	7.57±1.06	0.016*	7.41±1.00	7.27±0.93	0.508
	Mean change	0.58±0.60	0.72±0.80	0.348	0.78±0.78	0.51±0.60	0.081
HOMA-IR	Before treatment	2.36±1.05	2.98±2.33	0.115	2.55 [1.63,3.38]	1.98 [1.57,3.17]	0.333
	After treatment	1.68±0.75	1.74±0.96	0.764	1.66 [1.15,2.22]	1.18 [1.05,2.04]	0.138
	Mean change	0.69 [0.24,0.93]	0.67 [0.14,2.02]	0.395	0.65 [0.24,0.98]	0.77 [0.15,1.25]	0.653
Adiponectin (mg/L)	Before treatment	3.05 [1.54,5.29]	3.83[2.65,7.91]	0.042*	4.78±3.97	4.92±3.86	0.871
	After treatment	5.93 [2.46,9.28	5.6 [2.94,11.81]	0.471	7.40±4.76	6.56±5.48	0.464
	Mean change	−2.35±4.29	−2.03±4.18	0.737	−2.62±3.98	−1.63±4.46	0.299
TG (mmol/l)	Before treatment	1.89±0.86	1.92±1.02	0.913	1.96±0.85	1.83±1.05	0.542
	After treatment	1.46 [1.19,1.91]	1.61 [1.20,2.59]	0.431	1.62 [1.25,2.13]	1.38 [1.13,1.97]	0.216
	Mean change	0.25±0.76	−0.08±0.90	0.080	0.03±0.92	0.11±0.77	0.659
TC (mmol/l)	Before treatment	4.57±0.86	4.86±0.86	0.143	4.79±0.84	4.66±0.91	0.514
	After treatment	4.43±0.79	4.96±1.03	0.013*	4.82±1.03	4.61±0.88	0.343
	Mean change	0.14±0.92	−0.11±0.88	0.227	−0.03±0.97	0.05±0.82	0.700
LDL-C (mmol/l)	Before treatment	2.71 [2.30,3.24]	2.80 [2.40,3.32]	0.562	2.88±0.74	2.87±0.87	0.965
	After treatment	2.78 [2.30,3.11]	2.70 [2.33,3.34]	0.681	2.83±0.87	2.79±0.81	0.845
	Mean change	0.12±0.82	0.01±0.68	0.508	0.05±0.87	0.08±0.56	0.862
HDL-C (mmol/l)	Before treatment	1.24±0.30	1.32±0.29	0.198	1.26±0.28	1.32±0.31	0.358
	After treatment	1.27±0.26	1.38±0.35	0.118	1.31±0.35	1.37±0.28	0.383
	Mean change	−0.03±0.22	−0.06±0.23	0.599	−0.05±0.20	−0.05±0.25	0.976
BMI (kg/m^2^)	Before treatment	25.47±2.83	25.50±3.25	0.976	25.63±3.34	25.31±2.68	0.635
	After treatment	25.51±2.90	25.69±3.27	0.804	25.74±3.27	25.45±2.89	0.681
	Mean change	−0.04±0.91	−0.19±0.68	0.398	−0.11±0.84	−0.15±0.74	0.827
Waist circumference(cm)	Before treatment	90.54±7.19	90.74±10.05	0.919	91.00±9.34	90.22±8.27	0.698
	After treatment	89.57±7.34	90.70±9.55	0.550	90.38±9.21	89.97±7.91	0.836
	Mean change	1.00 −1.00,2.38]	0.00 −1.00,0.38]	0.149	0.00 [−1.00,2.00]	0.00 [−1.00,1.00]	0.506

FPG, fasting plasma glucose; HOMA-IR, homeostasis model assessment for insulin resistance; HbA1c, glycated hemoglobin; TG, triglyceride; TC, total cholesterol; LDL-C, low-density lipoprotein cholesterol; HDL-C, high-density lipoprotein cholesterol; BMI, body mass index.

Interestingly, in light of the fact that pioglitazone influences transcription of *ADIPOQ*, the two SNPs within the promoter region of the *ADIPOQ* gene (rs266729 and rs16861194), were not associated with a significant difference in the mean change of FPG, fasting insulin, HbA1c%, HOMA-IR, ADIPOQ, TG, TC, LDL-C, HDL-C, BMI, or WC between patients with rs266729 genotypes CC and CG + GG or between patients with rs16861194 genotypes AA and AG (data not shown).

Likewise, the mean changes in FPG, fasting insulin, HbA1c%, HOMA-IR, ADIPOQ, TG, TC, LDL-C, HDL-C, BMI, and WC after the pioglitazone treatment were not significantly different among the three SNPs within the exon 3 region of the *ADIPOQ* gene (rs2082940, rs1063538 and rs1063537; data not shown).

## Discussion

Individual differences in patient response to pioglitazone do exist, and many studies have focused on underlying genetic diversity at the *ADIPOQ* locus as the fundamental cause. Although there is agreement among studies performed by various investigators that *ADIPOQ* gene polymorphisms do influence the response to pioglitazone in T2DM patients, the results of individual studies are inconsistent [11], [12], [13]. The primary objective of this prospective intervention study was to determine whether genetic variation in the *ADIPOQ* gene might be the basis for the inconsistencies reported in response to pioglitazone treatment. The results revealed two major points. Firstly, the decrease in HbA1c% levels after the pioglitazone treatment was greater in those patients with the rs2241766 TG genotype than in patients with other genotypes. Secondly, where response was not evident by the HbA1c% criteria, significant changes in individual clinical correlates were associated with genotypes of individual polymorphisms. For example, the mean change in the FPG levels between the rs3774261 AA and AG + GG genotypes was significantly different. These findings may be clinically relevant to predict patients who will benefit most from pioglitazone treatment.

The most straightforward scenario for differences in pioglitazone response would be that the transcriptional activity of PPARγ, and thus plasma ADIPOQ levels, may differ based on sequence variability in the promoter region of the gene. Although pioglitazone has been shown to directly regulate transcription of the *ADIPOQ* gene through PPARγ, our data indicate ironically that there is no association between polymorphisms in the promoter region of the *ADIPOQ* gene (rs266729 and rs16861194) and response to the drug. The present findings confirm some of the previous findings reported on 80 northern Chinese Han patients with T2DM [10]. This study also revealed that there was no association between the promoter polymorphism rs16861194 and pioglitazone response. However, a weak association between a polymorphism in the second promoter region (rs266729) and HbA1c% values was found [10]. Specifically, rs266729 CG + GG versus CC was associated with a greater decrease in the HbA1c% value after the pioglitazone treatment (0.13% vs. 0.08%). Treatment protocols between the two studies differed slightly and may account for the association in their results: 10 weeks of treatment pioglitazone at a dose of 30 mg/day study [10] versus 12 weeks at a dose of 15 mg/day in this study.

The synonymous *ADIPOQ* SNP, rs2241766 (+45 T>G, glycine-to-glycine change), is one of the most common polymorphisms of the gene. Rs2241766 is in close proximity to the splice junction of intron 1 and exon 2 and may affect the splicing of the mRNA. Differences in response to pioglitazone may, thus, be the result of subsequent changes in ADIPOQ protein levels and activity. One of the interesting findings from our study is that the rs2241766 polymorphism was significantly associated with the response to pioglitazone in patients. Furthermore, response amongst these patients alone differed based on genotype. The response rate and mean decrease in HbA1c% values with pioglitazone treatment for patients with genotype TG was 52.94% and 1.15%, respectively. A response and mean decrease was also evident in HbA1c% values for patients with the TT genotype but to a lesser degree, 12.70% and 0.52%, respectively. These differences between the genotypes were statistically significant (*p*<0.001), indicating that a base pair difference of G to T may influence the efficacy of pioglitazone.

As one of the most common polymorphisms of the *ADIPOQ* gene, rs2241766 has been examined for association to treatment response by other investigators. In Iranian patients with T2DM, the rs2241766 polymorphism was not found to be associated with the response to pioglitazone [11], although overall Iranian patients did respond better to pioglitazone, 31.7% versus 21.25% in our study. The basis for the difference in response in Iranian patients is unknown and may be due to ethnic differences in the populations analyzed [14], [15]. One other possibility is that pioglitazone has physiological effects in addition to transcription mediated by PPARγ[16].

Rs2082940, rs1063538 and rs1063537, polymorphisms in exon 3 regions of the *ADIPOQ* gene, were also examined for associations with therapeutic efficacy of pioglitazone was investigated. However, there was no evidence of an association between response to pioglitazone and these polymorphisms. Additional studies including larger cohorts, as well as other common polymorphisms in *ADIPOQ* gene, will be helpful to further confirm our results.

A final analysis was performed to identify associations between polymorphisms and specific clinical phenotypes that might be obscured simply because of our definition of response to treatment. In this analysis, a unique, previously undescribed relationship of respone to pioglitazone with intron 2 SNPs, rs3774261, rs3821799, rs2241767, and rs1501299, emerged. Two clinical parameters, WC and insulin resistance, exhibited significant changes in patients with specific SNPs. The mean change in WC was significantly greater in treated patients with the rs3821799 genotype AA compared to those carrying genotypes AG + GG (P = 0.019). In contrast, the reduction in the insulin value of the rs3774261 genotype AG + GG patients was greater than for genotype AA (*p* = 0.030). Our results corroborate analysis performed on a cohort of Italian patients [17]. In the Iranian population, however, the putative relationship of the exon 2polymorphisms with WC was obscured. Only a non-significant association between the rs3821799 polymorphism and WC was observed [18].

The main trend of the future of medicine is personalized medicine, based in part on individual genotypes, in order to provide appropriate, safe, and economic use of a drug [19], [20]. This study provides novel data on the relationship between SNP in the *ADIPOQ* gene and the response to pioglitazone. Several caveats however still exist regarding the results. The major limitation of this study is the small sample size. Secondly, the study does not illuminate the molecular mechanisms by which genotype variants may influence the response to pioglitazone. Finally, the efficacy of pioglitazone is to a degree is dependent on environmental factors such as diet and exercise, and these factors were not addressed in this study. However, the results should stimulate further studies with a much larger sample size, perhaps encompassing more than simply the Chinese Han population, along with more strictly designed clinical and mechanistic studies.
